# Efficacy of Cassava Peel Extracts for the Removal of Heavy Metals from Hospital Sewage Sludge in Nigeria

**DOI:** 10.5696/2156-9614-9.23.190908

**Published:** 2019-08-06

**Authors:** Adedotun Timothy Adeolu, Solomon Olayinka Adewoye

**Affiliations:** 1 Department of Environmental Health Science, Kwara State University, Malete, Nigeria; 2 Department of Pure and Applied Biology, Ladoke Akintola University of Technology, Ogbomosho, Nigeria

**Keywords:** heavy metals, sewage sludge, *Aspergillus niger* fermentation extract, crude fermentation extract (CFE), cassava peels

## Abstract

**Background.:**

The use of strain-specific microbial fermentation in the production of organic acids for the removal of heavy metals from sludge has been extensively studied. However, there is scarce information on the use of microflora for fermentation.

**Objectives.:**

To assess the efficacy of cassava peel extracts for the removal of heavy metals from hospital sewage sludge in Nigeria.

**Methods.:**

A composite sewage sludge sample was collected from the University College Hospital sewage treatment plant in Ibadan, Nigeria and analyzed for heavy metals using standard methods. Aspergillus niger fermentation and crude fermentation extract were obtained from the cassava peels strain of Aspergillus niger and indigenous microflora, respectively. The experiment was carried out by adding 10 ml of the treatment to 3 g of each sludge sample (extracts and controls) at varied temperatures (room and elevated) and pH (3–5). The mixture was centrifuged after a contact time of 1–12 days at 1000 rpm for 1 hour. The filtrate was analyzed for heavy metals concentrations and compared with the standards. Data were analyzed using descriptive statistics and adsorption models.

**Results.:**

Mean heavy metal concentrations in the sludge were estimated for copper (2.22±0.2 mg/kg), zinc (52.3±0.1 mg/kg), chromium (1.46±0.1 mg/kg), nickel (5.6±0.01 mg/kg), and lead (1.9±0.1 mg/kg) and were below permissible limits. Optimum heavy metal removal for Aspergillus niger fermentation extract at room temperature was achieved on day 12 at pH 3.5 for zinc (74.5%), while optimum heavy metal removal at elevated temperature was achieved on day 9 at pH 3.0 for lead (79.3%). The optimum pH for crude fermentation extract lies between pH 3.0–4.5 for nickel (76.2%) at room temperature and chromium (76.6%) at elevated temperature.

**Conclusions.:**

Crude fermentation extract of cassava peel was found to be effective in removing heavy metals from sewage sludge. Therefore, its use could be adopted and promoted for removing heavy metals from sewage sludge to achieve safe disposal.

**Competing Interests.:**

The authors declare no competing financial interests.

## Introduction

Sludge is a solid residue generated during sewage treatment in industrial treatment plants. It may contain organic and inorganic compounds, non-essential trace elements, microorganisms, and the eggs of parasitic organisms.^1^,^2^ Increasing rates of urbanization and industrialization are responsible for the large volume of sludge being generated globally from domestic residences, industries, the agricultural and commercial sector, and institutions.^3^,^4^ Hospitals produce a significant volume of wastewater per day, which contains microorganisms, heavy metals, toxic chemicals and radioactive elements. According to Ekhaise and Omavwoya, differences in hospital sludge compared to municipal sludge are due to patient therapeutics (e.g. antibiotics, chemotherapy) which increase the content of organic matter, chemicals, metals, and pathogenic organisms.^5^ Heavy metals in hospital sludge originate from feces, paint, wear and tear of utensils and equipment, and radioactive materials from radiology departments.^5^,^6^

Sewage sludge is often used for agricultural applications due to its abundance of organic matter and nutrients.^7^ However, the presence of hazardous contents such as heavy metals, pathogenic organisms, and soluble salts limits its land application.^8^,^9^ Remediation of the heavy metals content of dewatered sludge is needed, since heavy metals are not degradable and once released into the soil environment, they have the potential to deteriorate the quality of soil, water (surface and underground), and human health and safety.^10^–^14^ Methods of extraction such as bioremediation, composting, stabilization of metals, and acid treatment have been employed in the removal of heavy metals from sludge.^15^–^18^ However, due to the complex matrix of sewage sludge and bonding nature of metals in organic solids, metals can only be satisfactorily solubilized under extreme acidic conditions achieved in high redox-potential environments using chemical leaching or bioleaching techniques.^7^

Inorganic chelating agents have been productively used in heavy metal removal as they form stable complexes over a broad pH range. However, their consistent use tends to decrease soil productivity and impair the physic-chemical structure of soils, thereby rendering soil unfit for further use.^11^,^16^ Other disadvantages of their use include persistence in the environment, high cost, and adverse health effects. For the bioleaching of metals from sewage sludge, organic acids are more promising than inorganic chelating agents, since extraction can occur in mildly acidic conditions (pH 3–4) and they are biodegradable and have low environmental impact. There is a need for a sustainable, environmentally-friendly, readily available, inexpensive, and efficient extractant for the removal of heavy metals.^19^–^24^

Nigeria is the largest cassava producer in the world, producing one-third more than Brazil and almost double the production capacity of Thailand and Indonesia, with an annual production of 49 million metric tons.^25^–^27^ Cassava peels represent a unique renewable carbon source and millions of tons are generated every day.^28^ Improper disposal of cassava peels includes littering the streets, dumping refuse in drains and causing blockages, or being discarded in bushy areas, becoming an eyesore, and creating breeding grounds for disease vectors and pathogens. The presence of cassava peels in the wet and heterogenous waste stream serves as an attractant and breeding site for houseflies, which are implicated in the transmission of typhoid and paratyphoid fevers, diarrhea, dysentery, cholera, gastroenteritis, amoebiasis, helminthic infestations, conjunctivitis, poliomyelitis, and other diseases that can spread by mechanical transmission.^29^

**Abbreviations**
*NESREA*: National Environmental Standards and Regulations Enforcement Agency
*WHO*: World Health Organization

The use of strain-specific microbial fermentation in the production of organic acids from agricultural wastes for bioleaching of heavy metals has been extensively studied.^30^–^32^ However, there is little information on the use of indigenous microflora for the bioleaching of heavy metals from hospital sewage sludge. The present study assessed the efficacy of strain-specific and indigenous microflora cassava peel extracts for the removal of heavy metals from hospital sewage sludge in Nigeria.

## Methods

**Figure 1:**
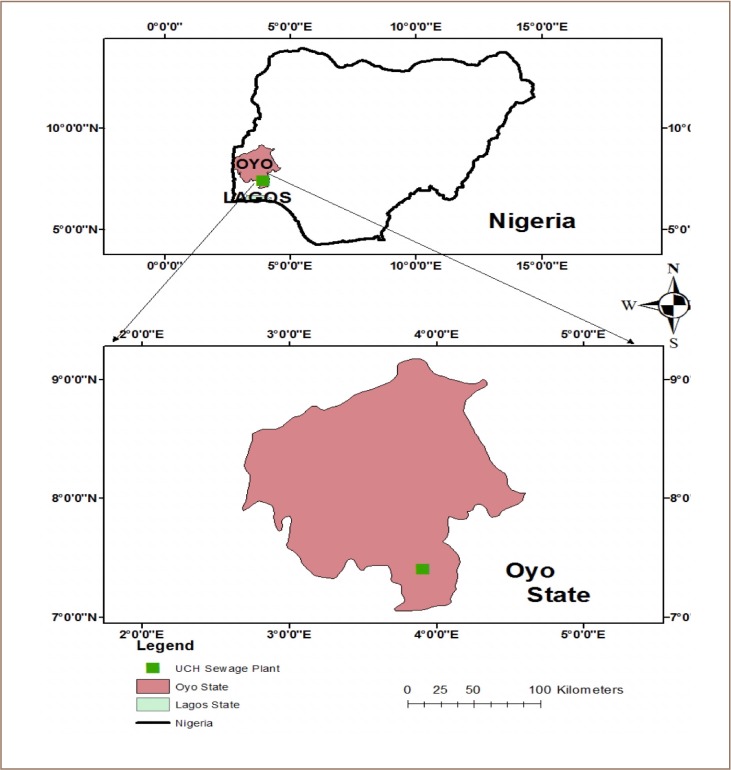
Study area

University College Hospital sewage treatment plant is located in Ibadan, Oyo State, Nigeria *(Figure 1).* The processes of the plant involve the conversion of organic matter into inorganic matter before discharging the treated effluent into the recipient environment (the Olojuoro River), while the sludge is processed into organic manure for agricultural purposes.

### Collection and analysis of dewatered sewage sludge

Composite sewage sludge was collected from the treatment plant, dried, homogenized, and analyzed for physico-chemical parameters (organic matter, total carbon, total nitrogen, and total phosphorus) using standards procedures.^9^,^33^ The gradient concentrations of heavy metals in the sludge were determined using an atomic absorption spectrophotometer (Buck Scientific Model 210 VGP).

### Isolation of bioleaching agent

The locally isolated citric acid-producing strain of Aspergillus niger was identified with the use of Czepak-Dox Agar. Aspergillus niger fermentation extract and crude fermentation extract were obtained from the fermentation of 3 grams of cassava peels for 13 days using the acids-producing strain of A. niger as inoculum and indigenous microflora in the cassava peels.^34^–^36^ These extracts were then used for bioleaching heavy metals from the sewage sludge, while commercial citric acid served as a control. The fermented materials were extracted with distilled water. Fermented sample extracts were collected every 48 hours for an estimation of citric acid over a period of 12 days. Citric acid produced by fermentation was estimated by the pyridine acetic anhydride method.^37^

### Extraction experiments

Heavy metal removal experiments were carried out to determine the efficacy of extracts (leaching agents or extractants) at varied optimum conditions (pH, temperature and contact time) using batch experiments as modified by Okareh and Enesi.^2^ The analyses were carried out at unadjusted and adjusted pH for all of the extractants. Adjustments of pH were carried out to determine the effect of progressive acidification on the removal of heavy metals from the sludge using 1 m hydrochloric acid. The extraction was carried out at varied pH levels (3–5) and contact times of 1, 3, 6, 9 and 12 days at ambient (28°C) and elevated temperature (45°C) for each extractant. For each extraction, the centrifuge tube containing 3 g of sieved sewage sludge was filled with 100 ml of the extractant. The tubes were stirred continuously on a rotary shaker at 150 rpm and centrifuged at 1000 rpm for 1 hour. The supernatant was filtered through a filter paper. The concentrations of heavy metals in the final solutions (filtrate) were determined by an atomic absorption spectrometer. The heavy metals concentrations in the sewage sludge were compared with the National Environmental Standards and Regulations Enforcement Agency (NESREA)^38^ and World Health Organization (WHO)^39^ permissible limits.

### Data management

Analyses of heavy metals were carried out in triplicate and data was subjected to descriptive statistical analysis using the Statistical Package for the Social Sciences (SPSS 21) software. In addition, the adsorption model was used to calculate the amount of metal ions leached *(Equation 1)* and percentage removal of heavy metals (removal efficiency) from the sewage sludge *(Equation 2).*

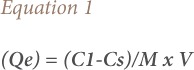


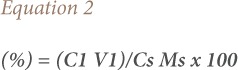
where Q_e_ is the metal uptake (mg/kg); C_1_ and C_s_ are the concentrations of the metal in extractants (mg/l) and sludge (mg/l), respectively, M_s_ is the mass of the sludge (kg) and V is the volume of the extractant (liter) (l).

## Results

The pH of the sewage sludge sample was 6.20±0.2, organic matter content was measured at 68.13±0.5%, total carbon was 35.13±0.5%, and the heavy metal contents (mg/kg) are shown in Table 1.

**Table 1:** Physico-Chemical Properties of Dewatered Sewage Sludge

**Parameter**	**Values**	**Standards**	
		**NESREA^38^**	**USEPA^39^**
PH	6.2± 0.2	6–9	6.5–8.5
Organic matter (%)	68.1± 0.5	NA	NA
Total carbon (%)	35.1± 0.5	NA	NA
Total nitrogen (%)	3.7± 0.4	NA	NA
Total phosphorus (%)	0.6± 0.1	NA	NA
Copper (mg/Kg)	2.2± 0.2	100	1500
Zinc (mg/Kg)	52.3±0.1	421	2800
Chromium (mg/Kg)	1.5±0.1	100	1200
Nickel (mg/Kg)	5.6±0.01	70	NA
Cadmium (mg/Kg)	ND	03	39
Lead (mg/Kg)	1.9± 0.1	164	300

The results are replicates of six samplings.

Abbreviations: ND, not detected; NA, not available.

### Efficacy of fermentation extracts for heavy metal removal

The percentage heavy metal removal of the fermentation extracts was measured at varied pH values and days. At room temperature, A. niger fermentation extract had the highest optimum heavy metal removal of 74.5% and 74.4% for zinc (Zn) and chromium (Cr), respectively. Crude fermentation extract had the highest optimum heavy metal removal of 76.2% and 74.8% for nickel (Ni) and Zn, respectively. Commercial citric acid had the highest optimum heavy metal removal of 75.5% and 73.6% for Zn and copper (Cu), respectively (*Table 2*). At elevated temperature, A. niger fermentation extract had the highest optimum heavy metal removal of 79.3% and 75.9% for lead (Pb) and Cu, respectively. Crude fermentation extract had the highest optimum heavy metal removal of 76.6% and 76.3% for Cr and Pb, respectively, while commercial citric acid had the highest optimum heavy metal removal of 79.2% and 77.9% for Zn and Ni, respectively (*Table 3*).

**Table 2:** Optimum Heavy Metal Removal at Room Temperature for Aspergillus niger Extract

**Heavy metals**		**Extractants**		

		Aspergillus niger fermentation extract	Crude fermentation Extract	Citric acid
**Cu**	Extraction	72.0	74.4	73.6
	Efficiency (%)			
	pH	3.5	3.5	3.0
	Contact time	3	9.0	12
**Zn**	Extraction	74.5	74.8	75.5
	Efficiency (%)			
	pH	3.5	3.5	4.0
	Contact time	12	12.0	6
**Cr**	Extraction	74.4	70.5	72.2
	Efficiency (%)			
	pH	3.0	4.5	3.5
	Contact time	9	9.0	3
**Ni**	Extraction	73.1	76.2	72.4
	Efficiency (%)			
	pH	3.0	4.0	3.0
	Contact time	3	12	1
**Pb**	Extraction	73.5	71.6	68.5
	Efficiency (%)			
	pH	3.0	3.0	3.0
	Contact time	12	12	12

**Table 3:** Optimum Heavy Metal Removal at Elevated Temperature for Aspergillus niger Extract

**Heavy metals**		**Extractants**		

		Aspergillus niger fermentation extract	Crude fermentation Extract	Citric acid
**Cu**	Extraction	75.9	75.6	72.6
	Efficiency (%)			
	pH	3.5	3.5	5.0
	Contact time	12	12	1
**Zn**	Extraction	75.2	75.1	79.2
	Efficiency (%)			
	pH	3.0	3.5	4.0
	Contact time	12	12	6
**Cr**	Extraction	75.6	76.6	75.2
	Efficiency (%)			
	pH	4.0	3.5	4.0
	Contact time	9	6	6
**Ni**	Extraction	74.1	75.2	77.9
	Efficiency (%)			
	pH	4.0	3.5	4.0
	Contact time	1	1	6
**Pb**	Extraction	79.3	76.3	74.9
	Efficiency (%)			
	pH	3.0	3.0	3.0
	Contact time	9	1	9

Optimum A. niger fermentation extraction occurred at elevated temperature with Pb (79.3%) and the lowest percentage extraction occurred for Ni (74.1%) as shown in Table 3. For crude fermentation, optimum extraction occurred at elevated temperature with Cr (76.6%) and the lowest percentage extraction occurred for Zn (75.1%) *(Table 3).*

### Effect of pH on heavy metal removal

**Figure 2:**
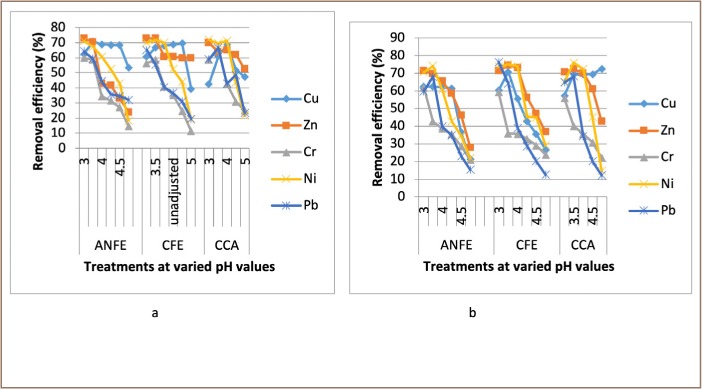
Effect of pH on heavy metal removal efficiency at (a) room temperature and (b) elevated temperature on day 1. Abbreviations: ANFE (Aspergillus niger fermentation extract); CFE (Crude fermentation extract); CCA (Commercial-grade citric acid (control)).

**Figure 3:**
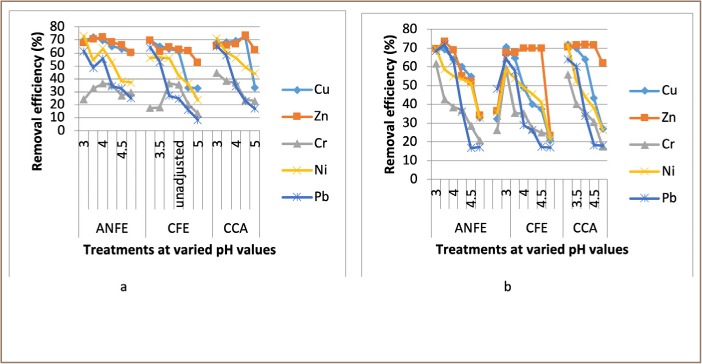
Effect of pH on heavy metal removal efficiency at (a) room temperature and (b) elevated temperature on day 3. Abbreviations: ANFE (Aspergillus niger fermentation extract); CFE (Crude fermentation extract); CCA (Commercial-grade citric acid (control)).

**Figure 4:**
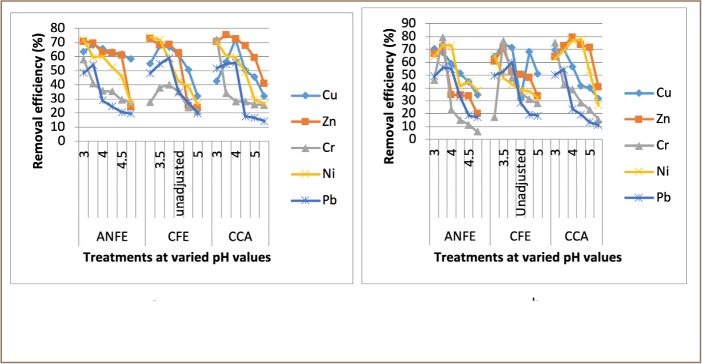
Effect of pH on heavy metal removal efficiency at (a) room temperature and (b) elevated temperature on day 6. Abbreviations: ANFE (Aspergillus niger fermentation extract); CFE (Crude fermentation extract); CCA (Commercial-grade citric acid (control)).

**Figure 5:**
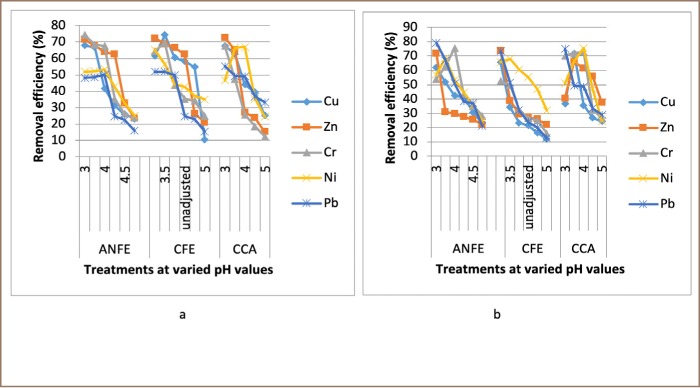
Effect of pH on heavy metal removal efficiency at (a) room temperature and (b) elevated temperature on day 9. Abbreviations: ANFE (Aspergillus niger fermentation extract); CFE (Crude fermentation extract); CCA (Commercial-grade citric acid (control)).

**Figure 6:**
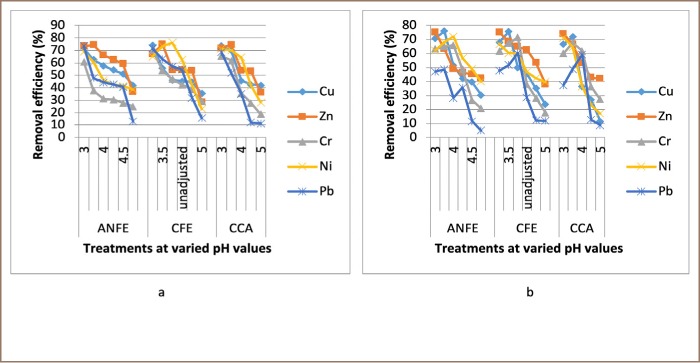
Effect of pH on heavy metal removal efficiency at (a) room temperature and (b) elevated temperature on day 12. Abbreviations: ANFE (Aspergillus niger fermentation extract); CFE (Crude fermentation extract); CCA (Commercial-grade citric acid (control)).

For some of the extractions, there was a corresponding increase in removal efficiency with an increase in pH from 3–4, followed by a decrease as pH increased towards 5. In a few instances, there was a decrease in removal efficiency with an increase in pH at varied temperatures and contact times, as presented in Figures 2–6.

### Effects of contact time on heavy metal removal

**Figure 7:**
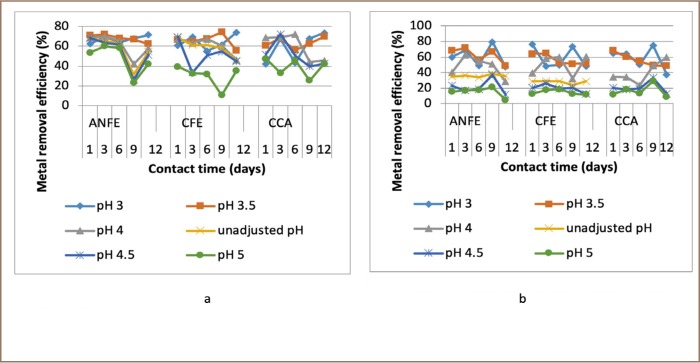
Effect of contact time on copper removal at (a) room and (b) elevated temperature. Abbreviations: ANFE (Aspergillus niger fermentation extract); CFE (Crude fermentation extract); CCA (Commercial-grade citric acid (control)).

**Figure 8:**
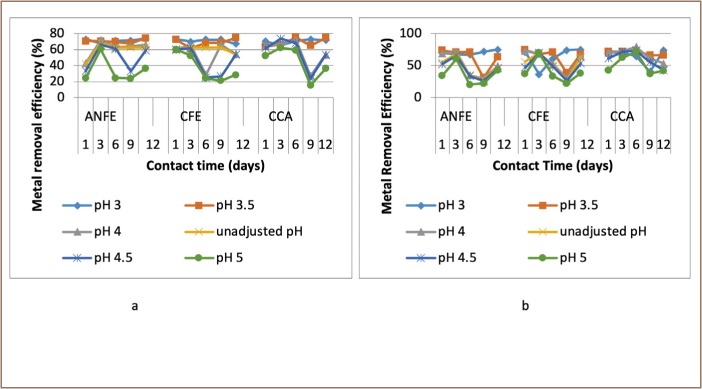
Effect of contact time on zinc removal at (a) room and (b) elevated temperature. Abbreviations: ANFE (Aspergillus niger fermentation extract); CFE (Crude fermentation extract); CCA (Commercial-grade citric acid (control)).

**Figure 9:**
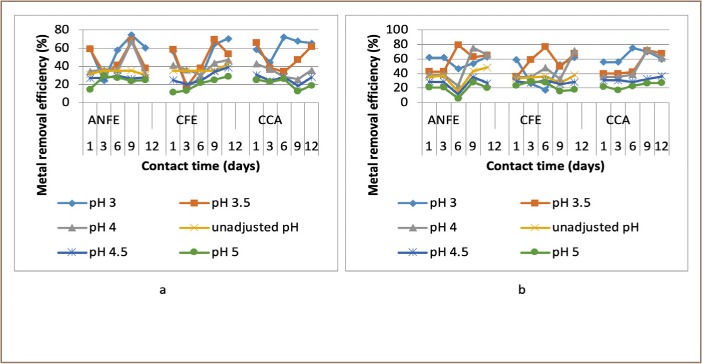
Effect of contact time on chromium removal at (a) room and (b) elevated. Abbreviations: ANFE (Aspergillus niger fermentation extract); CFE (Crude fermentation extract); CCA (Commercial-grade citric acid (control)).

**Figure 10:**
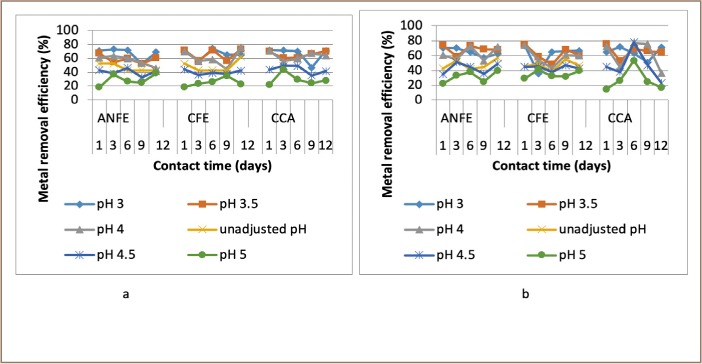
Effect of contact time on nickel removal at (a) room and (b) elevated temperature. Abbreviations: ANFE (Aspergillus niger fermentation extract); CFE (Crude fermentation extract); CCA (Commercial-grade citric acid (control)).

**Figure 11:**
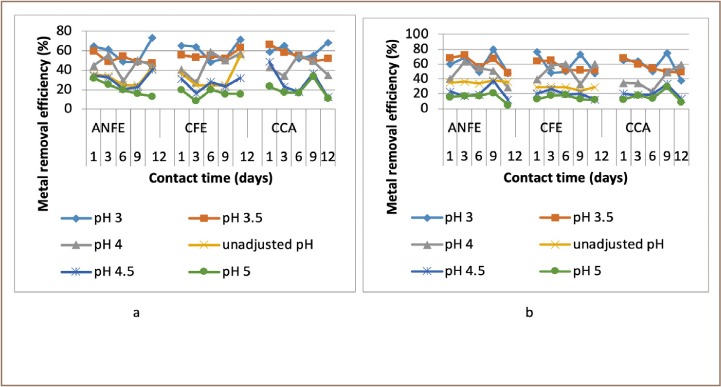
Effect of contact time on lead removal at (a) room and (b) elevated temperature. Abbreviations: ANFE (Aspergillus niger fermentation extract); CFE (Crude fermentation extract); CCA (Commercial-grade citric acid (control)).

Generally, there was a decrease of removal efficiency with increase in time from day 1–9. In a few instances, there was a corresponding increase in heavy metal removal efficiency as contact time increased and in others there was a downward trend in removal efficiency as contact time increased, as represented in Figure 7–11.

## Discussion

The average pH of the sludge sample was 6.20, which is within the WHO permissible limit of 6.5–8.5 and NESREA limit of 6.0–9.0; therefore, the discharge of sludge at a pH of 6.20 is acceptable to the receiving environment. The mean pH indicated that the dewatered sewage sludge was slightly acidic, as shown in Table 1. This finding was in line with that of Wuana *et al.*, who reported that the slightly acidic state of the sludge was within the range of agricultural soils.^40^ The importance of pH in metal solubility is well known, as it influences heavy metal adsorption, retention, and movement. The sewage sludge sample had a high percentage of organic matter in the forms of ammonium, nitrate, and other organic matter, as confirmed by Gaber *et al*.^41^ Organic matter influences the mobility of metals accumulated in surface layers in agricultural and urban soils.

While the heavy metals concentrations in the sludge were below the NESREA limits, concentrations of Zn and Cr were above the WHO limits, as shown in Table 1. The high concentrations of Zn in the present study were consistent with a study by Tolosana and Erhlich, which showed that effluent from medical institutions in South Africa had high levels of Zn and Cu.^42^ Kirchmann *et al.* linked the availability of Zn and Cu in sewage sludge to corrosion of plumbing systems, use of shampoos, and decomposition of paints.^32^ While the heavy metal concentrations were below the permissible limits for the safe disposal of sludge, their removal is still strongly recommended, since the accumulation of heavy metals at disposal sites constitutes a serious threat to the environment due to their bio-persistence, risk of groundwater contamination, and possible bioaccumulation in the food chain.^9^,^43^

### Efficacy of fermentation extracts for heavy metal removal

Optimum A. niger fermentation extraction occurred at elevated temperature with Pb and the lowest percentage extraction occurred for Ni. For crude fermentation, optimum extraction occurred at elevated temperature with Cr and the lowest percentage extraction occurred for Zn. These findings were consistent with those of Mingot *et al.* who reported that acid extracted amounts varied for each cation under examination and for each type of sludge.^44^

### Effects of treatment/extractant

Findings showed that A. niger fermentation extract was more effective in the extraction of heavy metals from sewage sludge at room temperature compared with commercial citric acid, except for Cu and Zn, as shown in Table 2. Extraction was more effective at elevated temperature compared with commercial citric acid, except for Zn and Ni. The extraction efficiency of crude fermentation extract was more desirable at elevated temperatures as it showed appreciably higher extraction efficiency for the heavy metals, except for Ni. It should be noted that Zn showed the greatest removal by the A. niger fermentation extracts at room temperature. This result is in agreement with the findings of Enesi, who reported that Zn is a more mobile element in soil and exhibits the same degree of mobility in sewage sludge.^43^

Lead showed the highest removal by the fermentation extracts at elevated temperature, as shown in Table 3. This result was in sharp contrast with a study by Okareh and Enesi, in which all of the extractants showed less than 20% Pb removal efficiency.^2^ The higher Pb removal efficiency of the fermentation extracts compared with the control indicates that the extracts formed more soluble complexes with Pb in the sewage sludge than the control. It should be noted that high removal of Cr was observed with A. niger fermentation extracts at both temperatures. The higher Cr removal efficiency of the A. niger fermentation extracts compared with citric acid indicated that extracts provide more binding sites or form more soluble complexes. Chromium-organic acid interactions are important for solubilization or binding of metals from the highly insoluble soil/sludge mineral phase. This is in contrast to the findings of Jakubus and Czekala who reported that, irrespective of oxidation degree, the dominant part of Cr (80–90%) was bound firmly and was difficult to dissolve in soil and sewage sludge.^45^

### Effects of pH on heavy metal removal

The optimum pH for A. niger lies between 3–4.0 for the removal of Pb (79.3%), while the crude fermentation extract lies between pH 3–4.5 for the removal of Ni (76.2%) at room temperature and Cr (76.6%) at elevated temperature *(Figures 2–6)*. The removal of heavy metals by progressive acidification for all of the analyzed metals depended on pH. These findings were consistent with previous studies which reported that pH is a dominant factor which influences the cation exchange capacity of the sludge, hence altering the redistribution and exchangeability of heavy metals in the sludge.^7^,^12^,^18^ As pH increased, the onset of the metal hydrolysis and precipitation commenced. When the pH of the extractant was increased from 3 to 4, there was a corresponding increase in deprotonation of the extractant's surface, leading to a decrease in hydrogen ions on the surface. This creates more negative charges on the extractant's surface, which favors extraction of positively charged species.

### Effects of contact time on heavy metal removal

Generally, there were initial increases in removal efficiency with corresponding increases in contact time, as depicted in Figure 7–11. The findings of the present study vary greatly from those of Stylianou *et al*., who reported that maximum heavy metal removal efficiency was attained in less than 24 hours using synthetic inorganic chelating agents.^7^ It should be noted that the initial increased rate was due to the availability of the uncovered surface area of the extractants since the kinetics depends on the surface area of the extractants. Extraction takes place at the more active binding sites. As these sites are progressively filled the more difficult the extraction becomes, as the extraction process tends to be more unfavorable.

### Management of cassava peel residues and treated sludge

The biosol (solid fraction of the fermented cassava peel) was added to the soil as recommended by Ubalua.^46^ The treated sludge (acidified) was dried, treated with lime, and applied to farmland as fertilizer and soil conditioner of high hygienic quality, as stated by Uriah *et al*.^47^

## Conclusions

Cassava peel wastes, which are often an environmental nuisance, could be used to produce resource materials (organic acids) which can be used to remove heavy metals of public health concern from industrial effluent/sewage sludge. Converting these wastes into organic acids has greatly helped to reduce its environmental risk and enhances effective waste management. The utilization of source segregated cassava peel waste for fermentation and the subsequent use of the fermentation extract for heavy metal removal from sewage sludge offers the dual benefit of environmental sanitation through waste material recycling and sustainable sewage sludge disposal through recycling of the cleaned sludge on farmlands. The removal of heavy metals from sewage sludge is a necessary step to achieving sustainable sludge treatment. In spite of the good heavy metal removal efficiency achieved in the most commonly used inorganic acid treatment method, factors such as cost, environmental sustainability, and technical adaptability of these methods are unattractive. The results of the present study to determine the efficacy of two fermentation extracts of cassava peel wastes in removing heavy metals from sewage sludge indicated that crude fermentation extract of cassava peel waste competed favorably with strain specific of Aspergillus niger in removing heavy metals from sewage sludge. In addition, the fermentation extract showed appreciably better heavy metal removal efficiency compared with commercial grade organic acids. Cassava peel wastes represent a cheaply available substrate associated with low fermentation process costs for obtaining crude fermentation extract, making this an attractive method for the removal of heavy metals from hospital sewage sludge waste.
